# Characterization of the complete plastome sequence of Korean endemic, *Cardamine glechomifolia* H.Lév. (Brassicaceae, Brassicales)

**DOI:** 10.1080/23802359.2024.2305394

**Published:** 2024-01-24

**Authors:** Sangjin Jo, Jinki Kim, Na-Rae Yun, Changyoung Lee, Sangho Choi, Soo-Yong Kim

**Affiliations:** a International Biological Material Research Center (IBMRC), Korea Research Institute of Bioscience and Biotechnology (KRIBB), Daejeon, Republic of Korea; b Operation Management Team, National Botanic Garden of Korea Native Plant, Gangwon-do, Republic of Korea; c Department of Botany, Honam National Institute of Biological Resources, Mokpo, Republic of Korea

**Keywords:** Plastome, Brassicaceae, *Cardamine*, *Cardamine glechomifolia*, Korean endemic

## Abstract

In this study, we report the complete plastome sequence of *Cardamine glechomifolia* H.Lév. 1913 (NCBI acc. no. OP894664). This plastome shows typical quadripartite structure. The plastome size is 154,307 bp, which consists of 84,015 bp large single-copy (LSC), 17,690 bp small single-copy (SSC), and 26,301 bp inverted repeat (IR) regions. The plastome contains 112 genes, including 78 protein-coding, 30 tRNA, and four rRNA genes. The *inf*A gene is pseudogenized. Sixteen genes contain one intron and two genes (*clp*P and *ycf*3) have two introns. The phylogenomic analysis conducted in our study reveals that the genus *Cardamine*, which encompasses *C. glechomifolia*, exhibits three distinct clades. In order to elucidate the interrelationship among the three clades, it is imperative to conduct additional investigations by augmenting the number of *Cardamine* samples.

## Introduction

*Cardamine* Linnaeus is a large genus of Brassicaceae and includes about 200 species (Christenhusz et al. 2017). They are distributed all over the world except Antarctica. *Cardamine glechomifolia* H.Lév. 1913 is a plant species exclusively found in Korea. The distribution of this species is primarily concentrated in Jeju Island and the southern coastal region of South Korea. The study of endemic plant species holds significance in the context of safeguarding national control and conservation of biological resources. In fact, complete plastome sequences of many species belonging to the genus *Cardamine* have been reported (Hu et al. 2015; Dann et al. 2017; Ren et al. 2017; Ru et al. 2020; Walden et al. 2020; Raman et al. 2021; Huang et al. 2022; Raman and Park 2022; Wang et al. 2022; Xu et al. 2022). However, for the conservation and management of endemic plants, we report for the first time the complete plastome sequence of *C. glechomifolia*, a Korean endemic plant.

## Materials and methods

**Figure 1.**
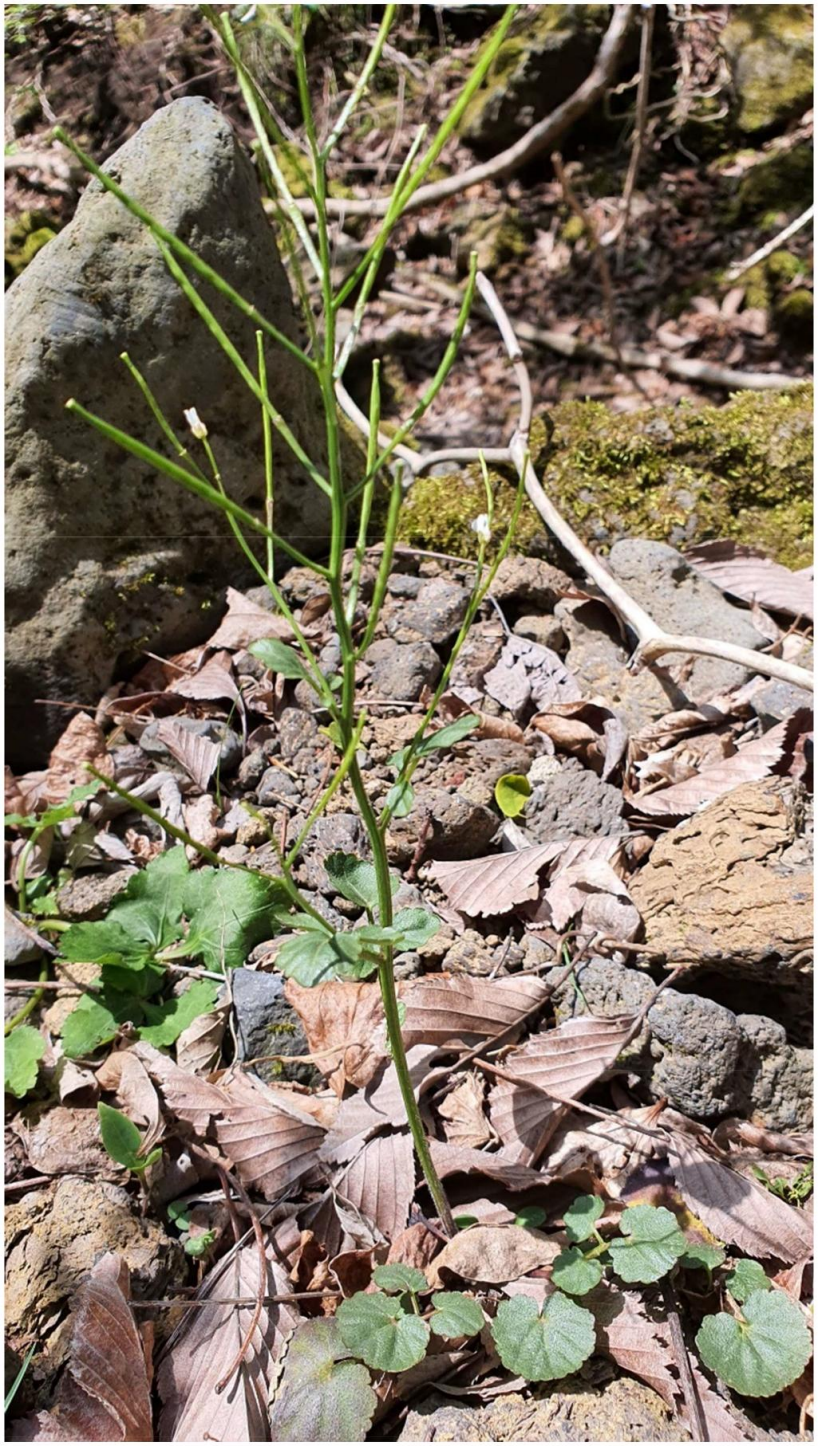
Field photo of *Cardamine glechomifolia* H.Lév. Species photo was taken by the author (Jinki Kim) in Jeju Island, South Korea, April 2020, without any copyright issues.

The study utilized freshly harvested leaves sourced from Jeju Island in South Korea (33°20′79.30″N and 126°22′48.30″E) at an altitude of 550 m (Figure 1). The voucher specimens were placed in the herbarium of the Korea Research Institute of Bioscience & Biotechnology (KRIB, acc. no. KRIB 0092706, Jin-Hyub Paik, jpaik@kribb.re.kr). Despite being an endemic species of Korea, *C. glechomifolia* does not possess the status of being endangered or protected. The study gained ethical approval from the Institutional Bioethics Committee of KRIBB.

The extraction of DNA from fresh leaves was performed using the DNeasy Plant Mini Kit manufactured by QIAGEN. The genomic DNAs are deposited in the International Biological Material Research Center (IBMRC acc. no. KRIB 0092706). Total raw reads (44,582,760) were generated using an Illumina NovaSeq platform (Illumina Inc., San Diego, CA). Total raw reads were assembled using NOVOPlasty 4.3.1.pl. (Dierckxsens et al. 2017). The complete plastome was annotated using the Geneious Prime v. 2022.2.2 (Biomatters Ltd., Auckland, New Zealand; Kearse et al. 2012), National Center for Biotechnology Information (NCBI) BLAST, Chloroplast Genome Viewer (CPGView) (Liu et al. 2023), and tRNAscan-SE 2.0 programs (Lowe and Eddy 1997). The circular plastome map was constructed by OrganellarGenomeDRAW (OGDRAW) (Greiner et al. 2019). For the phylogenomic analysis, we selected and downloaded 27 complete plastome sequences based on the APG IV system (APG IV 2016) from the NCBI database. The phylogenomic analysis was conducted on a dataset using RAxML v.8.2.12 on the CIPRES website, as described by Stamatakis (2014). The 28 plastome sequences, which had one inverted repeat (IR) sequence removed, were subjected to alignment using the MAFFT v.7.490 alignment programme in Geneious Prime v. 2022.2.2 (Biomatters Ltd., Auckland, New Zealand; Kearse et al. 2012). mVISTA analysis was performed to confirm the structural differences between *C. glechomifolia* and its relatives (Frazer et al. 2004).

## Results and discussion

**Figure 2.**
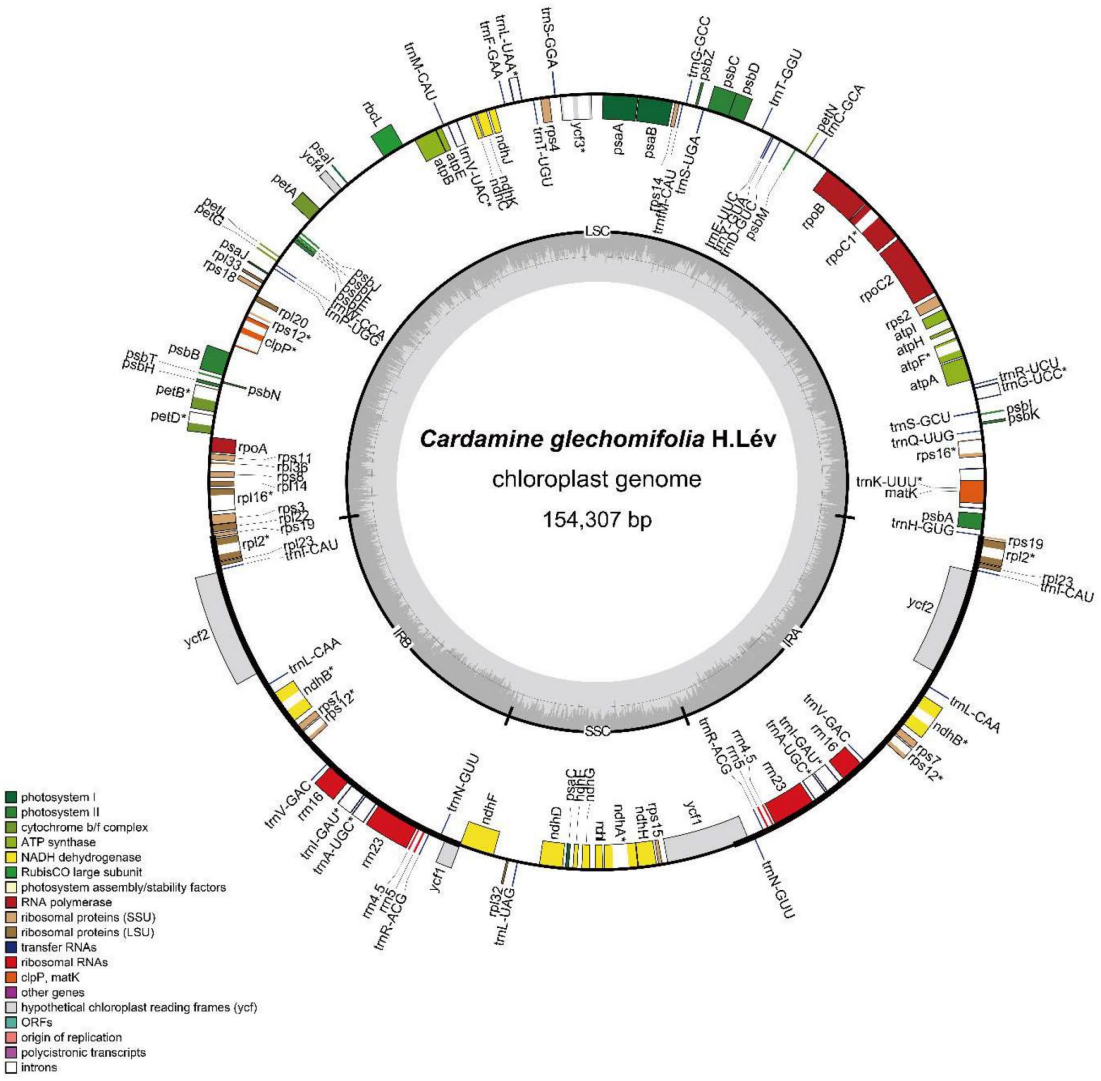
Complete plastome map of *Cardamine glechomifolia* H.Lév. The circular plastome map was constructed by OrganellarGenomeDRAW (OGDRAW). The genes inside and outside of the circle are transcribed in the clockwise and anticlockwise directions, respectively.

The length of the plastome of *Cardamine glechomifolia* is 154,307 bp. The structure of this entity conforms to a conventional quadripartite arrangement, as illustrated in Figure 2. It consists of four distinct sections: the large single-copy (LSC) region spanning 84,015 bp, the small single-copy (SSC) region spanning 17,690 bp, and two IR regions, namely IRa and IRb, each spanning 26,301 bp. The coverage of the plastome is 621.0×, as depicted in Figure S1. The plastome consisted of a total of 112 distinct genes, which can be further categorized into 78 protein-coding genes, 30 tRNA genes, and four rRNA genes. The IR regions contain duplicated copies of six protein-coding genes, seven tRNA genes, and four rRNA genes. The *inf*A gene can be classified as a pseudogene. The average guanine–cytosine (G–C) level is 36.3%. There are a total of 16 genes that possess a single intron, whereas two specific genes, namely *ycf*3 and *clp*P, exhibit the presence of two introns (Figures S2 and S3).

**Figure 3.**
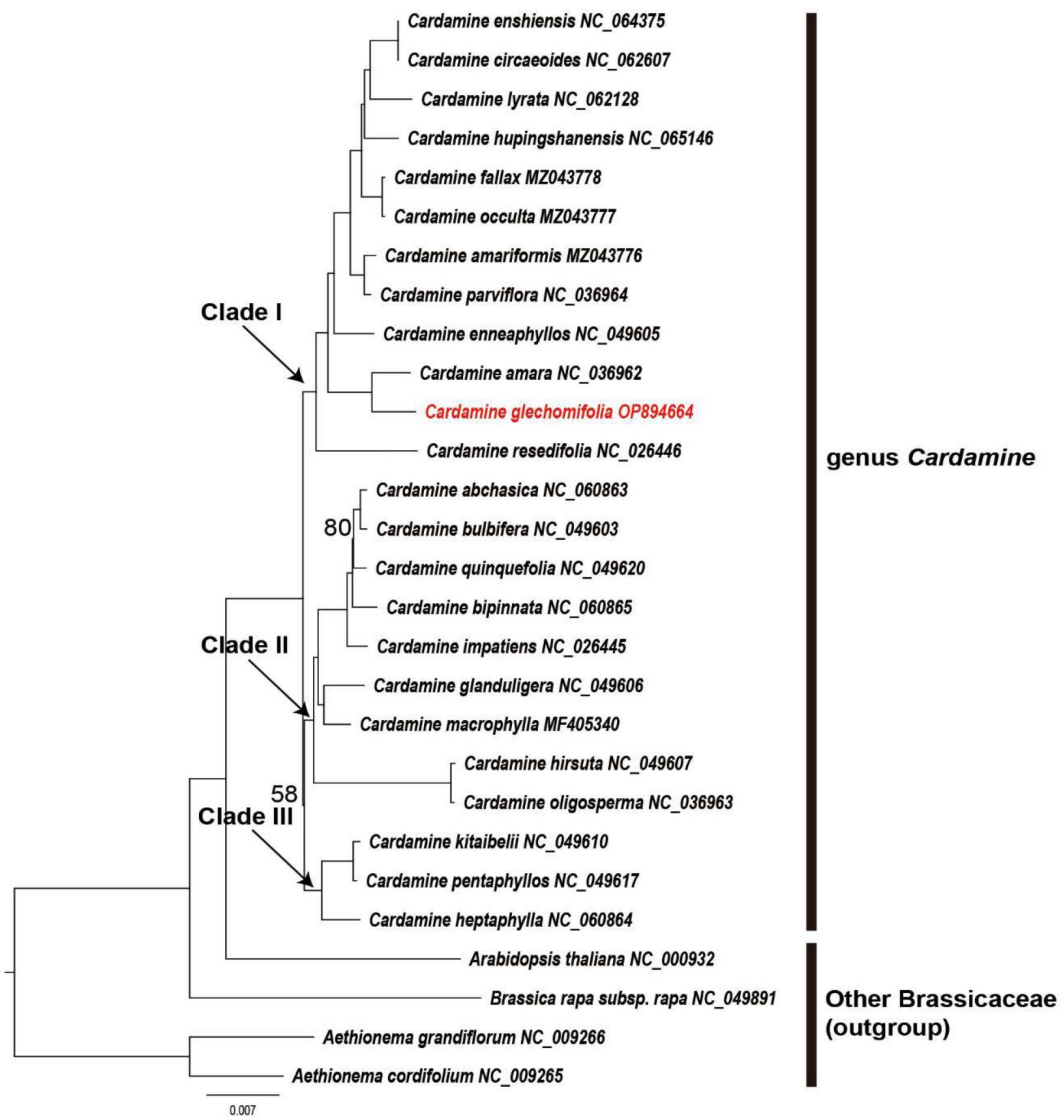
Maximum-likelihood (ML) tree based on 24 *Cardamine* and four other Brassicaceae plastome sequences (one IR sequences removed) as determined by RAxML(–ln *L =* 342550.479990). The values are displayed for nodes with bootstrap values less than 100%. No node value means the bootstrap value is 100%. The following sequences were used: *Aethionema cordifolium* (NC_009265.1) (unpublished), *Aethionema grandiflorum* (NC_009266) (unpublished), *Arabidopsis thaliana* (NC_000932) (Sato et al. 1999), *Brassica rapa* subsp. *rapa* (NC_049891) (Han et al. 2020), *Cardamine abchasica* (NC_060863) (Ru et al. 2020)*, Cardamine amara* (NC_036962) (Dann et al. 2017), *Cardamine amariformis* (MZ043776) (Raman et al. 2021), *Cardamine bipinnata* (NC_060865) (Ru et al. 2020), *Cardamine bulbifera* (NC_049603) (Walden et al. 2020), *Cardamine circaeoides* (NC_062607) (Wang et al. 2022), *Cardamine enneaphyllos* (NC_049605) (unpublished), *Cardamine enshiensis* (NC_064375) (unpublished), *Cardamine fallax* (MZ043778) (Raman and Park 2021), *Cardamine glanduligera* (NC_049606) (Walden et al. 2020), *Cardamine heptaphylla* (NC_060864) (Ru et al. 2020), *Cardamine hirsuta* (NC_049607) (Walden et al. 2020), *Cardamine hupingshanensis* (NC_065146) (Huang et al. 2022), *Cardamine impatiens* (NC_026445) (Hu et al. 2015), *Cardamine kitaibelii* (NC_049610) (Walden et al. 2020), *Cardamine lyrata* (NC_062128) (Xu et al. 2022), *Cardamine macrophylla* (MF405340) (Ren et al. 2017), *Cardamine occulta* (MZ043777) (Raman and Park 2022), *Cardamine oligosperma* (NC_036963) (Dann et al. 2017), *Cardamine parviflora* (NC_036964) (Dann et al. 2017), *Cardamine pentaphyllos* (NC_049617) (Walden et al. 2020), *Cardamine quinquefolia* (NC_049620) (Walden et al. 2020), and *Cardamine resedifolia* (NC_026446) (Hu et al. 2015).

In order to ascertain the phylogenomic relationships within the genus *Cardamine* in the Brassicaceae, a maximum-likelihood tree was generated. This was achieved by utilizing a dataset consisting of 24 *Cardamine* plastome sequences, along with four additional Brassicaceae sequences (serving as outgroup). Notably, one of the IR sequences was excluded from the analysis. The resulting alignment had a length of 137,087 bp. The tree analysis reveals that the genus *Cardamine* has given rise to three distinct clades, with *C. glechomifolia* positioned inside clade I (Figure 3). *C. glechomifolia* is considered a sister species to *C. amara*, as supported by a bootstrap value of 100% (Figure 3). The complete plastome structure was compared with clade I group (11 species) where *C. glechomifolia* is located. As a result, no structural differences between them were found (Figure S4). In this study, it was observed that *Cardamine* clade II and clade III exhibited proximity. However, earlier phylogenetic studies on *Cardamine* indicated a close connection between clade II and clade I, as Raman and Park (2021) and Huang et al. (2022) reported. To elucidate the interrelationship among the three clades, it appears imperative to procure more samples and do further investigation.

## Conclusions

This paper presents the complete plastome sequence of *Cardamine glechomifolia*, a plant species endemic to Korea. The study of endemic plant species holds significance in the context of safeguarding national control and ownership of biological resources. The plastome exhibits the characteristic structure typically observed in angiosperms. Our results revealed a close relationship between *C. glechomifolia* and *C. amara* as they nested together in our phylogenomic tree. We believe that our study holds significant importance in the resolution of *Cardamine*’s confusing phylogenetic relationship.

## Supplementary Material

Supplemental Material

## Data Availability

The data that support the finding of this study are openly available in GenBank of NCBI at https://www.ncbi.nlm.nih.gov, reference number OP894664 for *Cardamine glechomifolia*. The associated BioProject, BioSample, and SRA numbers are PRJNA904913, SAMN31857534, and SRR22401985, respectively.

## References

[CIT0001] Christenhusz MJM, Fay MF, Chase MW. 2017. Brassicaceae. In: Plants of the world: an illustrated encyclopedia of vascular plants. Chicago: University of Chicago Press. p. 415–419.

[CIT0002] Dann M, Bellot S, Schepella S, Schaefer H, Tellier A. 2017. Mutation rates in seeds and seed-banking influence substitution rates across the angiosperm phylogeny. bioRxiv. 156398.

[CIT0003] Dierckxsens N, Mardulyn P, Smits G. 2017. NOVOPlasty: *de novo* assembly of organelle genomes from whole genome data. Nucleic Acids Res. 45(4):e18. doi:10.1093/nar/gkw955. 28204566 PMC5389512

[CIT0004] Frazer KA, Pachter L, Poliakov A, Rubin EM, Dubchak I. 2004. VISTA: computational tools for comparative genomics. Nucleic Acids Res. 32(Web Server Issue):W273–W279. doi:10.1093/nar/gkh458. 15215394 PMC441596

[CIT0005] Greiner S, Lehwark P, Bock R. 2019. OrganellarGenomeDRAW (OGDRAW) version 1.3.1: expanded toolkit for the graphical visualization of organellar genomes. Nucleic Acids Res. 47(W1):W59–W64. doi:10.1093/nar/gkz238. 30949694 PMC6602502

[CIT0006] Han R, Tian M, Zhang G, Shao D, Ren Y. 2020. Complete chloroplast genome sequence of turnip (*Brassica rapa* ssp. *rapa*): genome structure and phylogenetic analysis. Mitochondrial DNA B Resour. 5(3):3555–3557. doi:10.1080/23802359.2020.1829124. 33458239 PMC7782280

[CIT0007] Hu S, Sablok G, Wang B, Qu D, Barbaro E, Viola R, Li M, Varotto C. 2015. Plastome organization and evolution of chloroplast genes in *Cardamine* species adapted to contrasting habitats. BMC Genomics. 16(1):306. doi:10.1186/s12864-015-1498-0. 25887666 PMC4446112

[CIT0008] Huang S, Kang Z, Chen Z, Deng Y. 2022. Comparative analysis of the chloroplast genome of *Cardamine hupingshanensis* and phylogenetic study of *Cardamine*. Genes. 13(11):2116. doi:10.3390/genes13112116. 36421792 PMC9690686

[CIT0009] Kearse M, Moir R, Wilson A, Stones-Havas S, Cheung M, Sturrock S, Buxton S, Cooper A, Markowitz S, Duran C, et al. 2012. Geneious Basic: an integrated and extendable desktop software platform for the organization and analysis of sequence data. Bioinformatics. 28(12):1647–1649. doi:10.1093/bioinformatics/bts199. 22543367 PMC3371832

[CIT0010] Liu S, Ni Y, Li J, Zhang X, Yang H, Chen H, Liu C. 2023. CPGView: a package for visualizing detailed chloroplast genome structures. Mol Ecol Resour. 23(3):694–704. doi:10.1111/1755-0998.13729. 36587992

[CIT0011] Lowe TM, Eddy SR. 1997. tRNAscan-SE: a program for improved detection of transfer RNA genes in genomic sequence. Nucleic Acids Res. 25(5):955–964. doi:10.1093/nar/25.5.955. 9023104 PMC146525

[CIT0012] Raman G, Park KY, Park SJ. 2021. The complete chloroplast genome of an endemic plant to Korea, *Cardamine amaraeformis* Nakai.: genome structure and phylogenetic analysis. Mitochondrial DNA B Resour. 6(9):2725–2726. doi:10.1080/23802359.2021.1955769. 34471692 PMC8405106

[CIT0013] Raman G, Park SJ. 2021. Complete chloroplast genome features and phylogenetic implications of *Cardamine fallax* (O. E. Schulz) Nakai. Mitochondrial DNA B Resour. 6(9):2722–2724. doi:10.1080/23802359.2021.1950067. 34471691 PMC8405073

[CIT0014] Raman G, Park SJ. 2022. Structural characterization and comparative analyses of the chloroplast genome of Eastern Asian species *Cardamine occulta* (Asian *C. flexuosa* With.) and other *Cardamine* species. Front Biosci (Landmark Ed). 27(4):124. doi:10.31083/j.fbl2704124. 35468683

[CIT0015] Ren T, Yang Y, Wang J, Zhang R, Liu ZL. 2017. Characterization of the complete chloroplast genome sequence of *Cardamine macrophylla* (Brassicaceae). Conserv Genet Resour. 10(4):627–630. doi:10.1007/s12686-017-0880-4.

[CIT0016] Ru Y, Schulz R, Koch MA. 2020. Successful without sex – the enigmatic biology and evolutionary origin of coralroot bittercress (*Cardamine bulbifera*, Brassicaceae). Perspect Plant Ecol Evol Syst. 46:125557. doi:10.1016/j.ppees.2020.125557.

[CIT0017] Sato S, Nakamura Y, Kaneko T, Asamizu E, Tabata S. 1999. Complete structure of the chloroplast genome of *Arabidopsis thaliana*. DNA Res. 6(5):283–290. doi:10.1093/dnares/6.5.283. 10574454

[CIT0018] Stamatakis A. 2014. RAxML version 8: a tool for phylogenetic analysis and post-analysis of large phylogenies. Bioinformatics. 30(9):1312–1313. doi:10.1093/bioinformatics/btu033. 24451623 PMC3998144

[CIT0019] Walden N, German DA, Wolf EM, Kiefer M, Rigault P, Huang X-C, Kiefer C, Schmickl R, Franzke A, Neuffer B, et al. 2020. Nested whole-genome duplications coincide with diversification and high morphological disparity in Brassicaceae. Nat Commun. 11(1):3795. doi:10.1038/s41467-020-17605-7. 32732942 PMC7393125

[CIT0020] Wang R, Deng Z, Luo Y. 2022. The complete chloroplast genome and phylogenetic analysis of *Cardamine circaeoides* Hook. f. et Thoms., 1861 (Brassicaceae). Mitochondrial DNA B Resour. 7(11):1964–1967. doi:10.1080/23802359.2022.2141081. 36406822 PMC9668279

[CIT0021] Xu X, Yao X, Zhang C, Xia P. 2022. Characterization of the complete chloroplast genome sequence of *Cardamine lyrata* Bunge (Brassicaceae). Mitochondrial DNA B Resour. 7(6):936–937. doi:10.1080/23802359.2022.2079106. 35692644 PMC9176338

