# Supplementary material for: Characterization of the complete plastome sequence of Korean endemic, Cardamine glechomifolia H.Lév. (Brassicaceae, Brassicales)
Source: Mitochondrial DNA B Resour. 2024 Jan 24;9(1):133–7. doi: 10.1080/23802359.2024.2305394 (PMC10810622; doi:10.1080/23802359.2024.2305394)
Supplement: Supplemental Material [file TMDN_A_2305394_SM7085.pdf]

Content:

Figure S1. The coverage of the complete plastome sequence of Korean endemic, *Cardamine glechomifolia*.

Figure S2. Schematic map of the cis splicing genes in the plastome of *Cardamine glechomifolia.*

Figure S3. Schematic map of the trans splicing genes in the plastome of *Cardamine glechomifolia.*

Figure S4. Comparion of 11 *Cardamine* plastome structure using *C. glechomifolia* as base reference. The purple regions indicate conserved exons and pink regions indicate conserved non-coding sequences (CNS). The Y-axis represents percent identity ranging from 50% to 100%.

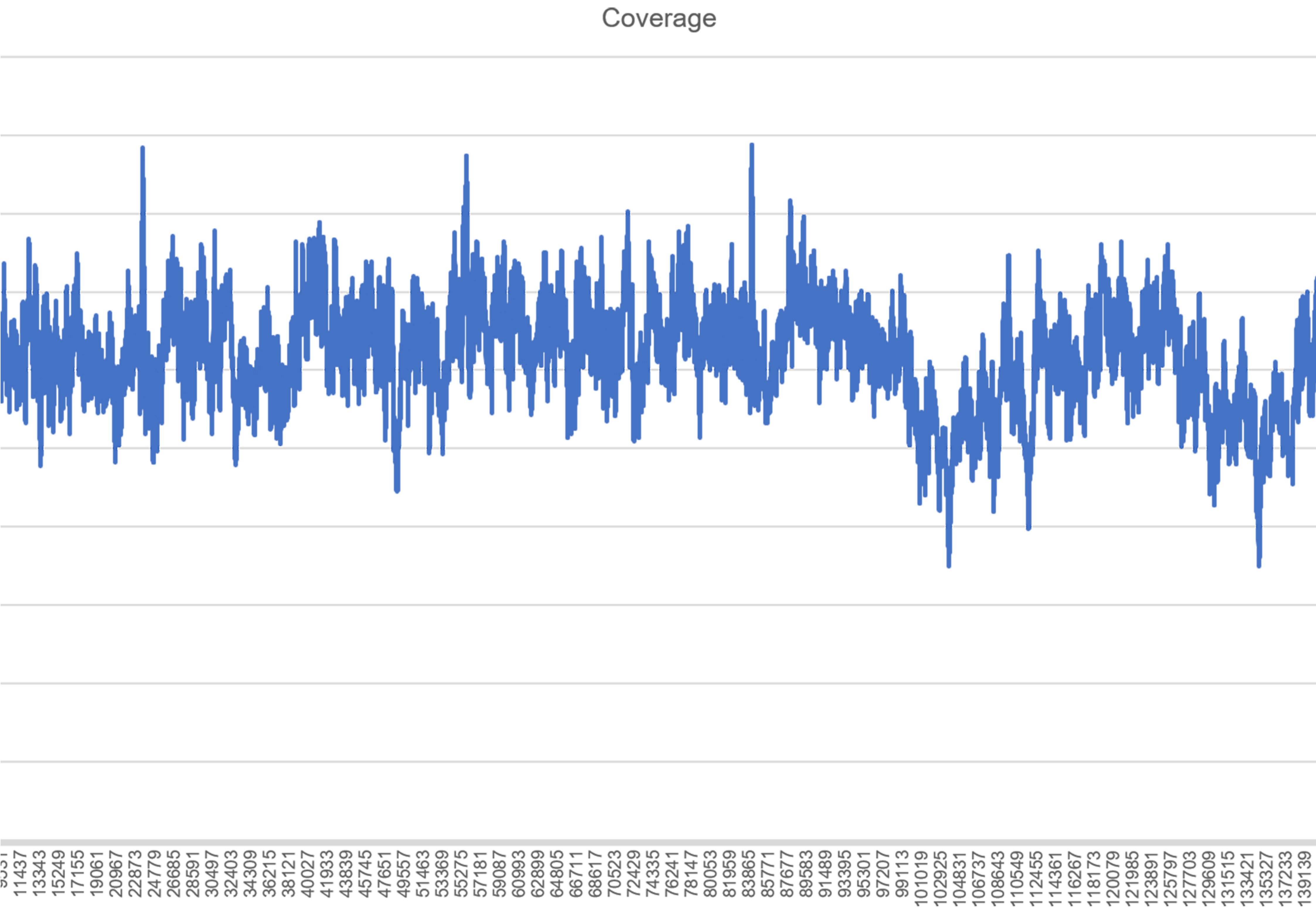
Coverage
11437
13343
15249
17155
19061
20967
22873
24779
26685
28591
30497
32403
34309
36215
38121
40027
41933
43839
45745
47651
49557
51463
53369
55275
57181
59087
60993
62899
64805
66711
68617
70523
72429
74335
76241
78147
80053
81959
83865
85771
87677
89583
91489
93395
95301
97207
99113
101019
102925
104831
106737
108643
110549
112455
114361
116267
118173
120079
121985
123891
125797
127703
129609
131515
133421
135327
137233
139139

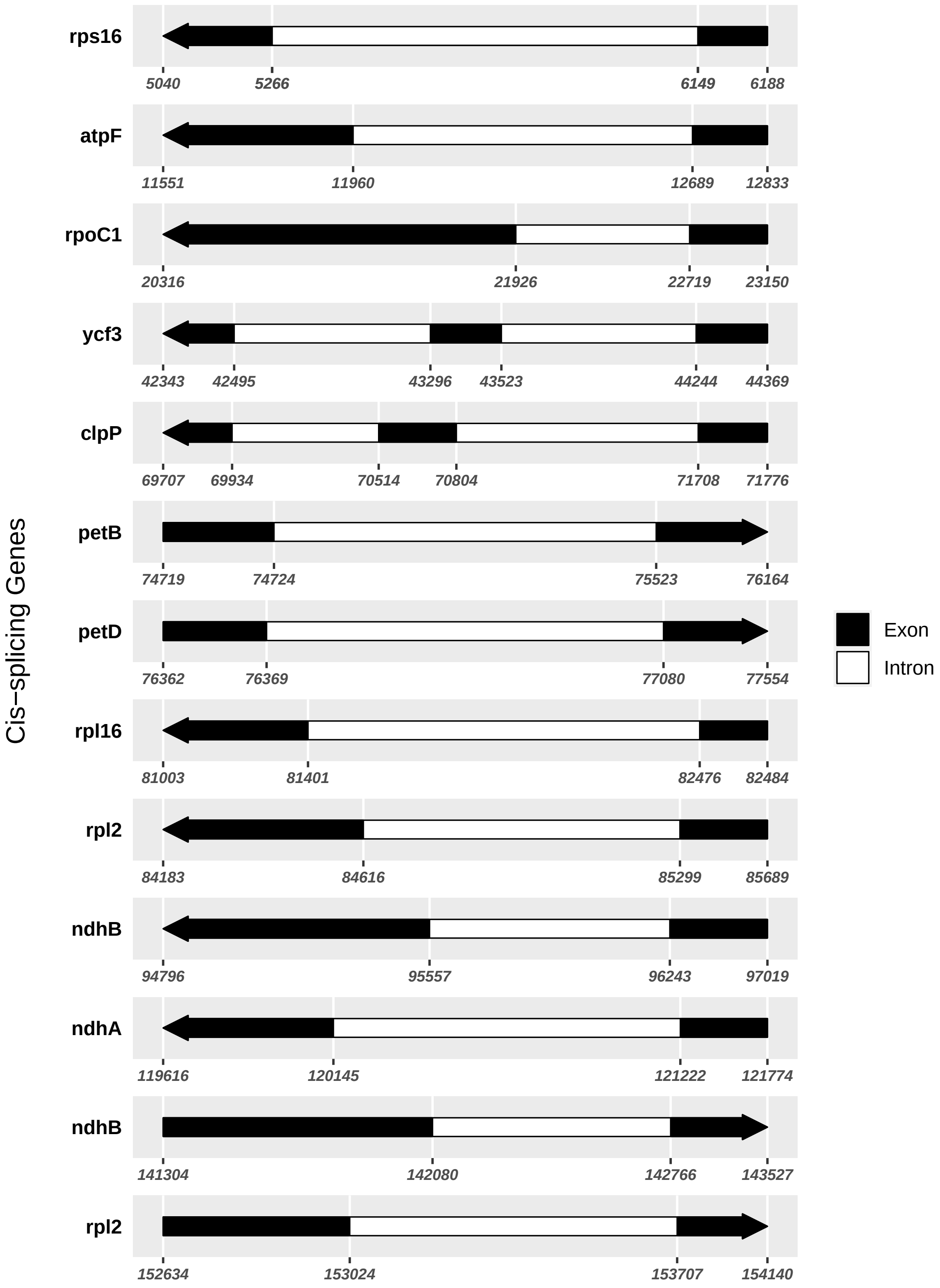

Cis−splicing Genes
rps16
5040
5266
6149
6188
atpF
11551
11960
12689
12833
rpoC1
20316
21926
22719
23150
ycf3
42343
42495
43296
43523
44244
44369
clpP
69707
69934
70514
70804
71708
71776
petB
74719
74724
75523
76164
petD
76362
76369
77080
77554
rpl16
81003
81401
82476
82484
rpl2
84183
84616
85299
85689
ndhB
94796
95557
96243
97019
ndhA
119616
120145
121222
121774
ndhB
141304
142080
142766
143527
rpl2
152634
153024
153707
154140
Exon
Intron

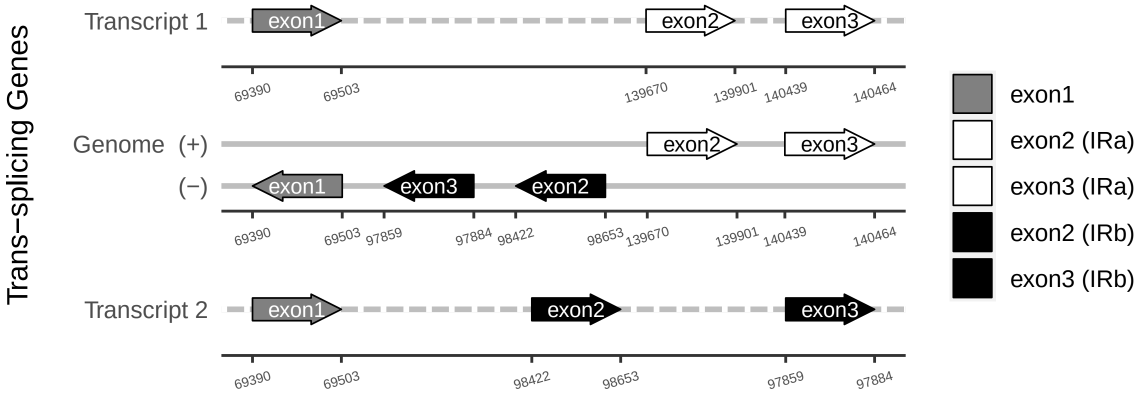

Trans−splicing Genes
Transcript 1
exon1
exon2
exon3
69390
69503
139670
139901
140439
140464
Genome (+)
(−)
exon2
exon3
exon1
exon3
exon2
69390
69503
97859
97884
98422
98653
139670
139901
140439
140464
Transcript 2
exon1
exon2
exon3
69390
69503
98422
98653
97859
97884
exon1
exon2 (IRa)
exon3 (IRa)
exon2 (IRb)
exon3 (IRb)

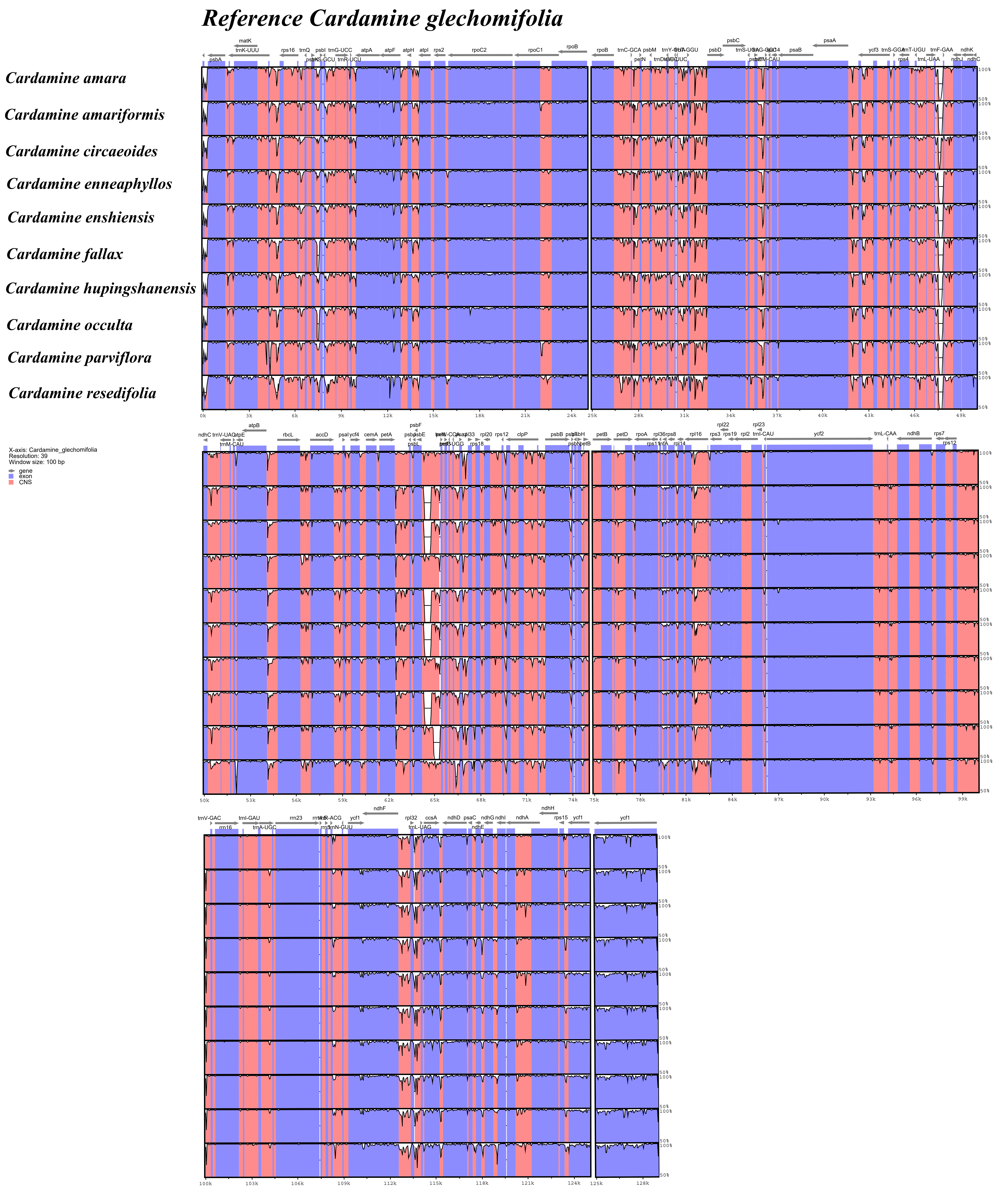
Reference Cardamine glechomifolia
Cardamine amara
Cardamine amariformis
Cardamine circaeoides
Cardamine enneaphyllos
Cardamine enshiensis
Cardamine fallax
Cardamine hupingshanensis
Cardamine occulta
Cardamine parviflora
Cardamine resedifolia
X-axis: Cardamine_glechomifolia
Resolution: 39
Window size: 100 bp
gene
exon
CNS
